# Study of Research and Development Processes through Fuzzy Super FRM Model and Optimization Solutions

**DOI:** 10.1155/2015/141946

**Published:** 2015-03-03

**Authors:** Flavius Aurelian Sârbu, Monika Moga, Gavrilă Calefariu, Mircea Boșcoianu

**Affiliations:** Faculty of Technological Engineering and Industrial Management, Transilvania University, Colina Universitatii No. 1 Corp A, 500036 Brașov, Romania

## Abstract

The aim of this study is to measure resources for R&D (research and development) at the regional level in Romania and also obtain primary data that will be important in making the right decisions to increase competitiveness and development based on an economic knowledge. As our motivation, we would like to emphasize that by the use of Super Fuzzy FRM model we want to determine the state of R&D processes at regional level using a mean different from the statistical survey, while by the two optimization methods we mean to provide optimization solutions for the R&D actions of the enterprises. Therefore to fulfill the above mentioned aim in this application-oriented paper we decided to use a questionnaire and for the interpretation of the results the Super Fuzzy FRM model, representing the main novelty of our paper, as this theory provides a formalism based on matrix calculus, which allows processing of large volumes of information and also delivers results difficult or impossible to see, through statistical processing. Furthermore another novelty of the paper represents the optimization solutions submitted in this work, given for the situation when the sales price is variable, and the quantity sold is constant in time and for the reverse situation.

## 1. Introduction

R&D (research and development) is the systematic and creative activity initiated to enhance the volume of knowledge, including those on human being and culture and using them for new applications.

The R&D function highlights the importance of research and development for social progress, being an intensive factor that can lead to spectacular leaps in national wealth and the hierarchy of nations as in [1].

The result of research and development is a unique product obtained with great effort and expense, and therefore in this case the resource requirements cannot be determined from the beginning.

The importance of the research and development function in the activity of an organization is evidenced by the fact that one of the defining features of modern economies is continuing consolidation and proliferation of innovative firms as in [2].

To compensate the lack of detailed statistics in this field (R&D) at regional level, in Romania, a good alternative is to carry out a survey that can investigate the current state of R&D at the regional level in Romania by assessing deficits, using a questionnaire and the FRM model.

This paper contributes to the elaboration of solutions for the improvement of regional research and development through FRM model, useful to all entities (companies, organizations, and institutions) concerned with basic, applied research and experimental development.

Another important aspect of this paper is given by the optimization solutions presented for firms, namely, the one suitable when the sales price is variable and the quantity is constant in time and reverse.

To maintain their position in the market industrial enterprises, as those from the field of services, are bound to continue improvement of products and technologies.

Considering these aspects we grouped the paper into five chapters. In chapter two, we present general aspects related to the method using Super Fuzzy Matrices. Chapter three is entitled generalities about the FRM model, while chapter four presents an overview on the method of Super Fuzzy FRM model used to evaluate the answers received from the respondents of the research in Romania.

In chapter five we present optimization solutions for companies; then we present the solution when the sales price is variable and the quantity is constant followed by the solution when the sales price is constant and the quantity is variable.

## 2. General Aspects Related to Using Super Fuzzy Matrices

Proposed by the Polish mathematician Jan Łukasiewicz (inventor of Polish notation) an early type of fuzzy logic has existed since 1920. His system allowed the extension of the truth value of a sentence to all real numbers in the range [0, 1].

A number in this range was interpreted as the possibility that the sentence is considered to be true or false. These researches led to the theory of possibility, a technique of reasoning in terms of inaccuracy.

Many different theories and tools have been proposed in the realm of decision making, in order to handle this issue, such as probability; however, after Rodriguez et al. (2012) in many cases, uncertainty is not probabilistic in nature but rather imprecise or vague as in [3].

Some methods had been developed for the traditional multiattribute decision making (MADM) problem for performing outranking of alternatives as in [4, 5]. One of the first among these methods was ELECTRE method discussed by Roy (1996), Roy and Berlier (1972), and Huang and Chen (2005) in [6–8].

Furthermore, similar methods like TOPSIS presented in [9–11] and VIKOR discussed by Opricovic and Tzeng (2004, 2007) in [11, 12] are based on the idea that optimal vector-valued alternative(s) should have the shortest distance from the positive ideal solution and farthest distance from the negative ideal solution.

There exists a series of works based on applying interval and fuzzy techniques to extend AHP as in Lu et al. (2007) or in Enea and Piazza (2004), TOPSIS as in Wang and Elhag (2006), Lu et al. (2007), and Li (2007), and VIKOR as in Liu and Wang (2011) and Park et al. (2011) to fuzzy environment as in [4, 13–17].

Originally introduced by Zadeh (1965), the fuzzy set theory is one of the most efficient decision aid techniques providing the ability to deal with imprecise and vague information.

Lotfi Zadeh in 1965 extended the possibility theory into a formal system of mathematical logic and also discussed ways of working with nuanced terms of natural language. This tool of representation and manipulation of nuanced terms is called fuzzy logic. Traditional logic considers that an object can belong to a crowd. Fuzzy logic allows a more flexible interpretation of the concept of belonging. Thus, many items may belong to a crowd within varying degrees as in [18].

**Figure 1:**
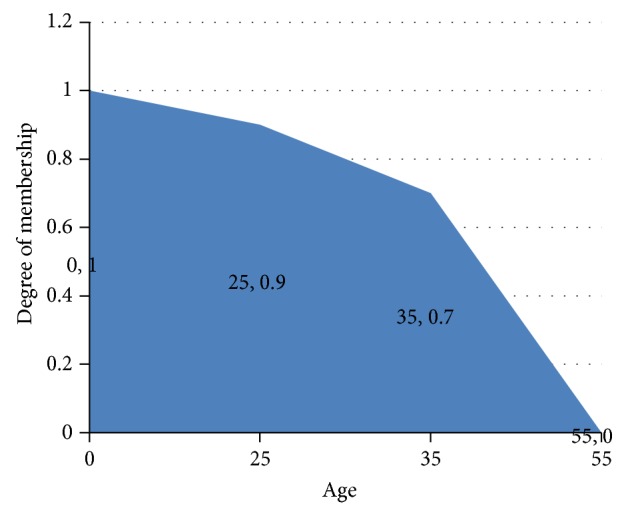
The plot of the example.

For example, if we consider the multitude of young people, a 15-year-old child is certainly young, while a 65-year-old-person certainly is not. But a 35- or 45-year-old man? Let us say for the example mentioned above, with the linguistic variable young, that we have the universe of discourse *X* = {0,25,35,55} and the following membership function: *A* = 0/1 + 25/0,9 + 35/0,7 + 55/0, meaning a 25-year-old person belongs to the set of young men in a proportion of 90% and a 35-year-old in a proportion of 70%, while a 55-year-old does not belong to the set (degree of membership is 0). These are plotted as in Figure 1.

On a universe of discourse several fuzzy subsets can be defined. For example, for the universe of ages of people, we can define subsets of young, old, or middle aged people. These subsets can intersect (this is highly recommended). The same person will belong to the subset of young people with 70% and to the subset of older people with 30%.

Often, people cannot accurately characterize well the numerical information using forms: almost and around 100. In fuzzy set theory, these numbers can be represented as fuzzy subsets of the set of real numbers. A fuzzy number *A* is a fuzzy set of the set of real numbers, with a convex and continuous membership function and limited support.

Furthermore, to cope with imperfect and imprecise information whereby two or more sources of vagueness appear simultaneously, the traditional fuzzy set shows some limitations; therefore, it has been extended into several different forms, as the intuitionistic fuzzy set, the interval-valued intuitionistic fuzzy set, the type 2 fuzzy set, the type *n* fuzzy set, the fuzzy multisets, and so on. These extensions are based on the rationale that it is not clear to assign the membership degree of an element to a fixed set discussed by Torra and Narukawa (2009) and Torra (2010) in [19, 20].

## 3. Generalities about the FRM Model

To study mainly the problem related to R&D for the first time the three new models (super column fuzzy relational maps, mixed super row fuzzy relational maps, and super fuzzy relational maps) have been introduced.

Therefore in the following we describe super column fuzzy relational maps model. Suppose we have some *n* sets of experts, forming some *n* distinct category of groups based on education or age or profession and so on. We can describe this model as a multiset of expert's model: we have sets of experts that is not a multiexpert model but multiset of expert's model. Thereby we have *n* sets of experts and each set may contain different special features. However the only common factor is that they all agree to work upon the same problem with the same set of attributes as in [21–24].

In the following we present what are the domain and the range spaces of this super column fuzzy relational maps model. The domain space is a fuzzy super mixed row vector relating all the *n* sets of experts who have worked with the model, while the range space of this model has state vectors which are simple row vectors taking its entries from the set {0,1} as in [23, 24].

Next we define and describe the new row super fuzzy relational maps. We have *n* sets of attributes related with a problem which is divided into different sets and some *n* experts view it and give their opinion. Each of these *n*-sets view the problem from a different angle. Therefore at each stage the problem is viewed in a very different way. That is why we construct a single model so that the hidden pattern is obtained as in [23–25].

Furthermore we proceed on to describe the super FRM model that comes handy when several sets of experts work with different sets of attributes. We have some problem *P* at hand and we have *n* sets of experts *N*_1_, *N*_2_, *N*_3_, and *N*_*n*_ where each *N* is a set of experts, *i* = 1,2, *n*. We also have some *p* sets of attributes: we have *M*_1_, *M*_*p*_ sets of attributes. Suppose we have some experts work on some sets, say, *M*_*i*_, *M*_*k*_,…, *M*_*t*_, 1 ≤ *i*, *k*,…, *t* ≤ *n*. Similarly some other sets of experts want to work with *M*_*s*_, *M*_*r*_, *M*_*l*′_,…, *M*_*m*_, 1 ≤ *s*, *r*, *l*, *m* ≤ *n* where we may have some of the set of attributes *M*_*i*_, *M*_*k*_,…, *M*_*t*_ which may be coincident with the set of attributes *M*_*s*_, *M*_*r*_,…, *M*_*m*_ as in [23, 24, 26].

We cannot apply any of the fuzzy models to this. Therefore we use a new model by combining the two models, presented above. We describe the fuzzy super matrix as follows: let the *N*_*i*_th set of experts give their opinion using the *M*_*j*_th set of attributes, and let *P*_*ij*_ denote the connection FRM matrix with the *N* set of attributes forming the part of the domain space and *M*_*j*_ attributes forming the range space. This is true for 1 ≤ *i* ≤ *n* and 1 ≤ *j* ≤ *p* as in [23, 24, 27–30].

**Figure 2:**
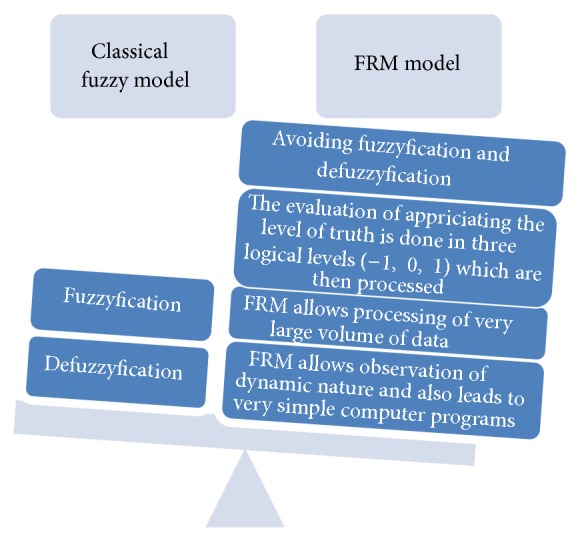
Comparison between the Classical Fuzzy Model and the FRM Model.

To sum up the abovementioned parts we try to compare the classical Fuzzy model and the FRM model by identifying the main differences between them (Figure 2).

## 4. An Overview on the Method of Super Fuzzy FRM Model Used in the R&D Research for the Central Region of Romania

In our study based on questionnaire we wanted to identify the existence and the importance of R&D activity in the Central Region of Romania.

The study had 12 samples, meaning that at the first step 12 firms and organizations with R&D activity responded to the 19 questions; here we have to mention that in our Super Fuzzy FRM model the following questions are included: Q1, Q2, Q3, Q5, Q6, Q7, Q13, Q15, Q17, Q18, and Q19.

R1–R12 represent the firms and organizations from the Central Region of Romania with R&D activity, which forms the sample of our study.

Q1: In which county is the headquarter of your company, institution, or organization located? This question has six response options: the six counties that form this region, represented in our model by Q1.1–Q1.6.

Q2: Since when is your enterprise, organization, or institute operating? This question has two response options: before or after 1989, represented in our model by Q2.1 and Q2.2.

Q3: What has your company, organization, or institute between 2010 and 2012 introduced? This question has four response options: a new product, a new manufacturing process, a new organization method, or a new marketing approach, represented in our model by Q3.1–Q3.4.

Q5: What is the area where you operated between 2010 and 2012? This question has five response options: locally, regionally, nationally, at the EU level, or at other markets (outside the EU), represented in our model by Q5.1–Q5.5.

Q6: Please indicate the number of employees in 2012. This request has four response options: between 0 and 9, 10 and 49, 50 and 249, or more than 250 employees, represented in our model by Q6.1–Q6.4.

Q7: Did your enterprise, institute, or organization in 2012 have R&D activity? This question has the following response options: yes or no, represented in our model by Q7.

Q13: Have you purchased in 2012 R&D from outside your business? This question has the following response options: yes or no, represented in our model by Q13.

Q15: Please choose a scientific field specific for the R&D activity of your company from the list below for 2012. This request has six response options: natural and exact sciences, engineering and technological sciences, medical and health sciences, agricultural sciences, social and economic sciences, or human sciences, represented in our model by Q15.1–Q15.6.

Q18: The employed personnel in 2012 in R&D was classified as. This request has three response options: researchers, technicians, or others, represented in our model by Q18.1–Q18.3.

Q19: Do you intend to carry out R&D activity in 2013? This question has the following response options: yes or no, represented in our model by Q19.

We give choice for the 12 respondents to select any response option from the total of 37; then we use fuzzy model in general and super fuzzy mixed FRM model in particular to analyze the options given by the fuzzy super matrix in Table 1.

Having the respondent's opinion in a super fuzzy connection FRM matrix we find the super hidden pattern for two vectors given by us.

(1) To study the effect of Q7, did your enterprise, institute, or organization in 2012 have R&D activity, let

 
*X* = (0000000000000000000001000000000000000)
 
*X*∗*M* = (101111011111) = *Y*
 
*Y*∗*M* ^*T*^ = (141103557622135101621 10 22513124298745 10) = *Z*, *Z*_*i*_ < 10 = 0, *Z*_*i*_ ≥ 10 = 1
 
*Z* = (0000000000000000000001000000000000001)
 
*Z*∗*M* = (202222022222) = *Z*_1_, *Z*_1_ ≤ 0 = 0, *Z*_1_ > 0 = 1
 
*Z*_1_ = (101111011111) = *Y*. Thus the supper hidden pattern is given by the binary pair {(0000000000000000000001000000000000001), (101111011111)}.

(2) To study the effect of Q19, do you intend to carry out R&D activity in 2013, let

 
*X* =  (0000000000000000000000000000000000001)
 
*X*∗*M* = (101111011111) = *Y*
 
*Y*∗*M* ^*t*^ = *Z*_1_
 Or *X* =  (000000000001)
 
*X*∗*M* ^*T*^ = (0000010111100010000101001000000101001) = *Y*
 
*Y*∗*M* = (63396625697 13) = *X*_1_, *X*_1_ < 13 = 0, *X*_1_ ≥ 13 = 1, *X*_1_ = (000000000001) = *X*.

Thus the supper hidden pattern is given by the binary pair {(0000010111100010000101001000000101001), (000000000001)} showing a very strong influence on the system.

Hence, we have to mention that when 0 is used in the model it means the negation of the statement; for example, for question 7 that represents the first vector of our study only the respondents 2 and 7 gave a negative response, meaning that they did not have R&D activity in 2012. Or for question 19, representing the other vector, the same respondents (2 and 7) gave negative response, meaning that they do not intend to have R&D activity in 2013, marked with 0 in our model, proving an intense correlation between the two vectors. If we consider the other side of these two questions, we have to mention that the majority of the respondents had R&D activity in 2012 and look forward to have it in 2013 as well.

It appears that this super hidden pattern is composed of two vectors: the all response variants vector, and the vector of the respondents.

The response variants vector contains the number 0 at the position in which respondents have a major negative expression (i.e., they express negative answers for the question) and the number 1 at the corresponding position of responses that expressed a majority of positive positions (i.e., they express positive answer to the question).

The vector of the respondents characterizes how respondents answer question Q7 and contains the number 0 at the position to which respondents give a negative answer and the number 1 at the position related to respondents giving an affirmative answer.

## 5. Study of R&D Processes through Optimization Solutions

As shown by the statistical data, to maintain their position in the market industrial enterprises, as those from the field of services, are bound to continue improvement of products and technologies as in [31–33].

Those who do not invest enough in R&D will have an increasingly smaller market and their profit will be also becoming smaller. Such companies, in the medium or long term, are bound to decline then to extinction. Before the extinction the decline is manifested, which can be presented in three ways as in [34, 35].

The first way is that, to maintain the rhythm of sales (quantity sold per unit time), the company is obligated by the mechanism of supply and demand to sell at increasingly lower prices. Thus it will sell at distances increasingly higher than the equilibrium point of supply and demand curves. In the short term, the company is in decline.

The second way is that the company does not agree to sell at lower prices, in which case, by the market mechanisms, the company will start selling increasingly smaller amounts in unit of time. Of course, without intervention by innovation, the company is in a situation similar to that described in the first bad way.

It is also possible to manifest the third mode, which is the simultaneous expression of the first two of the above. It is likely that both price and quantity sold decrease occures simultaneously. In this case the situation will be even worse.

Out of the three modes, in this paper the first two will be treated with the following assumptions.

1. During the stability of the sales price and the quantity sold it is considered that they are rigorously constant.
2. During the period of decline (acceptable in short term), it is considered that since the outbreak, it is linear.
3. Through research and innovation the market value of the products and the quantities sold are recovered, to the same level as before the decline in the short term. Basically we are in the situation of a firm that has a market maintaining strategy. The problem, of course, can be approached from the perspective of a growth strategy. The issue of a long-term decline strategy, of course, makes sense only in case of onerous deals, without ethics.

In the following we determine the period, the number of R&D cycles in a given period, and the maximum amount of profit.

### 5.1. Modelling the Situation When the Sales Price Is Variable and the Quantity Sold Is Constant in Time

**Figure 3:**
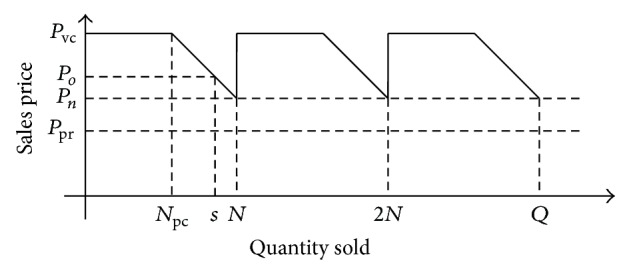
Modelling the situation when the sale price is variable and the quantity sold is constant in time.

It is considered that the company sales have the variance of sales price given by Figure 3, where

 
*N* is number of products sold after which innovation was introduced;
 
*N*_pc_ is number of products sold at constant prices;
 
*P*_vc_ is unit sales price constant until the selling of *N*_pc_ products;
 
*P*_*n*_ is unit sales price at *N* units sold;
 
*P*_pr_ is unit price at breakeven;
 
*C*_tu_ = total unit costs;
 
*C*_cd_ = cost of research and development for an innovation cycle (during the sale of *N* items);
 
*Q* = volume of production (the amount expected to be sold) during the strategic period (covered by the strategy of the company).

Dependence between the sales price at any position *P*_*o*_ at some *s* position of the range [0, *N*] will be

(1) Po=Pvcif  s∈[0,Npc]Pvc+Pvc−PnN−Npcs−Npcif  s∈Npc,N.

The report (*P*_vc_ − *P*_*n*_)/(*N* − *N*_pc_) characterizes the decrease rate of the sales price over the quantity sold *N*_pc_. It is, moreover, the slope that models the linear decrease of the price on (*N*_pc_, *N*]. Determining the slope means that the report is determined either by the past experience of the company, by using the results of other companies, or by marketing research. This report will be noted below, with *G*. Due to the linear decrease model of sales price within the range specified above, it follows that *G* is a constant parameter, which, as has been said, characterizes the slope in question.

Hence the dependence of *P*_*n*_ and *N* will be

(2) $$\begin{array}{c} P_{n}=P_{\text{vc}}-G\left(N-N_{\text{pc}}\right). \end{array}$$

**Figure 4:**
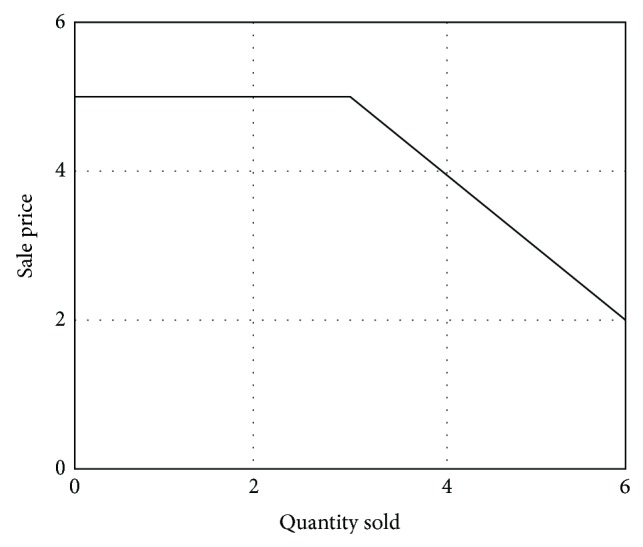
Graphical representation of relationship (1) in Matlab.

Graphically representing the relationship in Matlab, with generic data, deductible from the graphic, Figure 4 is obtained.

Figure 4 proves the correctness of relationship (2).

Dependence between the sales price *P* at any position *s* of the interval [0, *Q*], which encompasses *Q*/*N* intervals of type [0, *N*], will be

(3) Po=Pvc if  s−sN  N∈[0,Npc]Pvc+Pvc−PnN−Npcs−sNN−Npc if  s−sN  N∈Npc,N.

The expression [*s*/*N*] represents the integer (rounding to the nearest integer) of the relation between parentheses.

**Figure 5:**
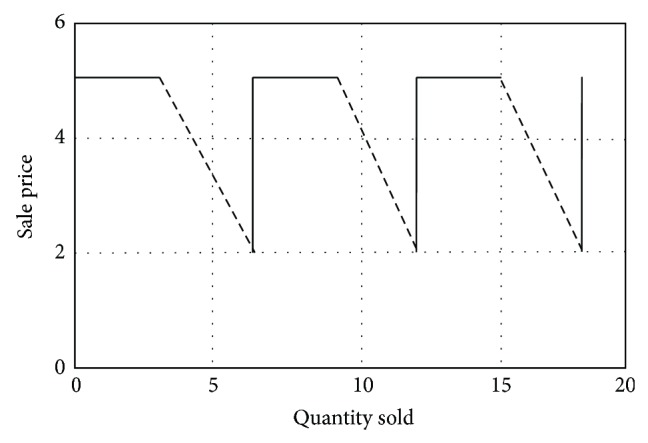
Graphical representation of relationship (3) in Matlab.

Similar to the previous case, simulating in Matlab relationship (3) with generic data, deductible from the graphic, Figure 5 is obtained.

To develop an accurate strategy at a time horizon corresponding to the sale of a quantity of *Q* products the total profit *Pr*⁡_*o*_ is calculated obtained during this period. This will be

(4) Pr⁡o=Npc·Pvc+N−NpcPvc−Pn2QN −CtuQ−CcdQN,

where *C*_tu_ is total production costs per unit of production; *C*_cd_ is the cost of R&D during the selling period of *N* products.

By ordering after *N* the formula of profit will be

(5) Pr⁡o=QPvc+GNpc−Ctu−GN2−GNpc2+2Ccd2N.

**Figure 6:**
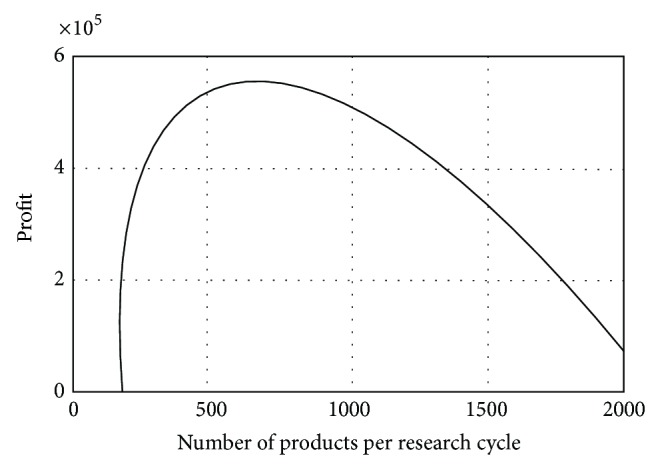
The dependence between the profit *Pr*⁡_*o*_ and the number of items sold after which innovation is introduced (Matlab).

The dependence between the profit *Pr*⁡_*o*_ and the number of items sold after which innovation is introduced can be seen in Figure 6, also in Matlab.

The value that leads to the maximum profit is obtained by derivation:

(6) $$\begin{array}{c} \frac{\partial {\mathrm{Pr}}_{o}}{\partial N}=Q\left(\frac{G{N_{\text{pc}}}^{2}+2C_{\text{cd}}}{2N^{2}}-\frac{G}{2}\right). \end{array}$$

By equaling to zero the number of products *N*_*p*max⁡_ after the selling of which, to achieve the maximum profit for the studied period (strategic), should be introduced as an innovation. This number will be

(7) $$\begin{array}{c} N_{P\max}=\sqrt{\frac{G{N_{\text{pc}}}^{2}+2C_{\text{cd}}}{G}}. \end{array}$$

Clearly, the maximum value of profit will be

(8)  Pr⁡o_max =QPvc+GNpc−Ctu−GNPmax⁡2−GNpc2+2Ccd2NPmax⁡.

To obtain the maximum profit depending on the initial data *N*_*P*max⁡_ is replaced and we get

(9) $$\begin{array}{c} {\mathrm{Pr}}_{o}\text{\_max}=Q\left(P_{\text{vc}}+GN_{\text{pc}}-C_{\text{tu}}-\sqrt{G\left(G{N_{\text{pc}}}^{2}-2C_{\text{cd}}\right)}\right). \end{array}$$

The number of RDI cycles during the strategic period (selling *Q* products) in order to have the maximum profit is given by

(10) $$\begin{array}{c} N_{\mathrm{cycle}}=\frac{Q}{N_{P\max}}. \end{array}$$

### 5.2. Modelling the Situation When the Sale Price Is Constant and the Quantity Sold Is Variable

**Figure 7:**
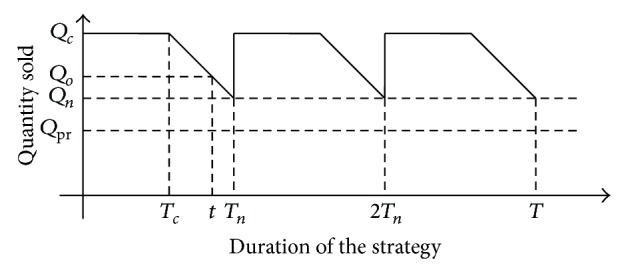
Modelling the situation when the sale price is constant and the quantity sold is variable.

It is considered that the company's sales have the following variation of quantity sold as given by Figure 7, where

 
*T*_*n*_ is moment of time at which an innovation is introduced;
 
*T*_*c*_ is moment of time until the quantity sold per unit time is constant;
 
*Q*_*c*_ is the level of constant sales in time until *T*_*c*_;
 
*Q*_*n*_ is the level of sales per unit time at the moment *T*_*n*_;
 
*C*_tu_ = total unit costs;
 
*C*_cd_ = cost of research and development in an innovation cycle (during the sale of *N* products);
 
*T* = duration of the strategy.

*Q*_*o*_ quantity sold per unit time, at some time *t* of interval [0, *T*_*n*_], will be

(11) Qo=Qcif  t∈[0,Tc]Qc+Qc−QnTn−Tct−Tcif  t∈Tc,Tn.

It is noted that *D* = (*Q*_*c*_ − *Q*_*n*_)/(*T*_*n*_ − *T*_*c*_) is the ratio which characterizes the decrease rate of the quantity sold beyond the moment in time *T*_*c*_. It is, moreover, the slope that shapes the linear decrease of the quantity sold per unit time interval (*T*_*c*_, *T*_*n*_]. As in the previous case, the determination of the ratio *D* is carried out either by the past experience of the company or by using the results of other companies or by marketing research. Considering the linear decrease model of the selling price within the range specified above, it follows that *D* is a constant parameter which characterizes the respective slope.

Hence the dependency of *Q*_*n*_ and *T*_*n*_ will be

(12) $$\begin{array}{c} Q_{n}=Q_{c}-D\left(T_{n}-T_{c}\right). \end{array}$$

The dependence of the quantity sold per unit time *Q*_*o*_ at any position *t* of the interval [0, *T*], which encompasses *T*/*T*_*n*_ intervals such as [0, *T*_*n*_], will be

(13) Qo=Qc if  t−tTn  Tn∈[0,Tc]Qc+Qc−QnTn−Tct−tTnTn−Tc if  t−tN  Tn∈Tc,Tn.

The expression [*t*/*T*_*n*_] represents the integer part (rounded to the nearest integer) of the relation between brackets.

It is found that the form of mathematical expressions is similar to the previous case (sales price variable and quantity sold constant in time): so the graphical check of the above relations is not necessary.

To develop a proper strategy on a time horizon *T* the total profit *Pr*⁡_*o*_ is calculated obtained during this period. This will be

(14) $$\begin{array}{c} {\mathrm{Pr}}_{o}=\left\{\left[Q_{c}\cdot T_{c}+\left(T_{n}-T_{c}\right)\frac{Q_{c}-Q_{n}}{2}\right]\left(Pv-C_{\text{tu}}\right)-C_{\text{cd}}\right\}\frac{T}{T_{n}}, \end{array}$$

where *C*_tu_ is total unit production costs of manufacture; *C*_cd_ is costs of research development in the period *T*_*n*_.

By ordering after *T*_*n*_ the formula of the profit will be

(15) Pr⁡o=TQcPv−Ctu+DtcPv−Ctu−DTn2Pv−Ctu  −DPv−CtuTc2+2Ccd2Tn.

**Figure 8:**
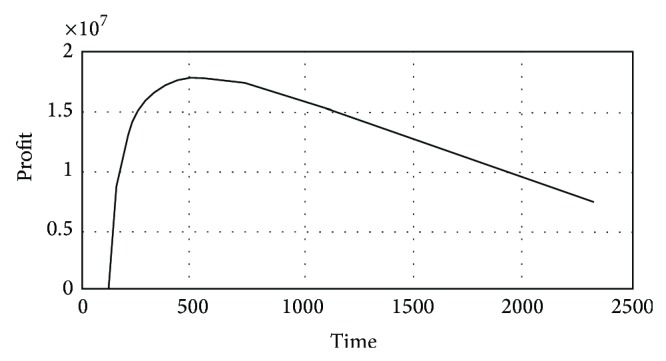
The dependence between the profit *Pr*⁡_*o*_ and the number of products sold after which innovation is introduced (Matlab).

The dependence between the profit *Pr*⁡_*o*_ and the number of products sold after which innovation is introduced can be seen in Figure 8, made in Matlab.

The value leading to maximum profit is obtained by derivation:

(16) $$\begin{array}{c} \frac{\partial {\mathrm{Pr}}_{o}}{\partial T_{n}}=T\left(\frac{D\left(Pv-C_{\text{tu}}\right){T_{c}}^{2}+2C_{\text{cd}}}{2{T_{n}}^{2}}-\frac{D\left(Pv-C_{\text{tu}}\right)}{2}\right). \end{array}$$

By equaling to zero the time interval *T*_*n*max⁡_ after the selling of which, to achieve the maximum profit for the studied period *T* (strategic), should be introduced as an innovation. This number will be

(17) $$\begin{array}{c} T_{n\max}=\sqrt{\frac{D\left(Pv-C_{\text{tu}}\right){T_{c}}^{2}\;+\;2C_{\text{cd}}}{D\left(Pv-C_{\text{tu}}\right)}}. \end{array}$$

Under these conditions, the maximum profit will be

(18) Pr⁡o_max=TQcPv−Ctu+DtcPv−CtuDTc2Pv−Ctu+2Ccd2Tnmax⁡  −DTnmax⁡2Pv−Ctu  −DTc2Pv−Ctu+2Ccd2Tnmax⁡.

To obtain the maximum profit depending on the initial data *T*_*n*max⁡_ is replaced and we get

(19) Pr⁡o_max=TQcPv−Ctu+DtcPv−CtuDPv−CtuTc2+2Ccd −DPv−CtuDPv−CtuTc2+2Ccd.

In order to obtain the maximum profit for the strategic period *T* the number of cycles of RDI (research-development-innovation) is given by

(20) $$\begin{array}{c} N_{\text{cicluri}}=\frac{T}{T_{n\max}}. \end{array}$$

## 6. Conclusion

To measure resources for R&D at the regional level in Romania and obtain primary data that is important in making the right decisions to increase the competitiveness and development based on an economic knowledge we carried out a survey based on questionnaire. Thus we obtained the R&D resources and funding sources as well as parameters specific for the employees within the R&D activity, available in the Central Region of Romania.

Then for the interpretation of the results we used the Super Fuzzy FRM model (as this theory provides a formalism based on matrix calculus, which allows processing of large volumes of information and also delivers results difficult or impossible to see, through statistical processing): we have at hand a problem *P* which is worked out by 12 respondents and they give their views on 11 questions with several response options, given by the fuzzy super matrix *M*, where *M*_*ij*_ *'*s are fuzzy matrices which correspond to the connection matrices of the FRM. *M* is known as the super dynamical system of the fuzzy super FRM maps. We wished to study the effect of two state vectors, *X* = (0000000000000000000001000000000000000) and (0000000000000000000000000000000000001), on the dynamical system *M*. We get *X*∗*M* = *Y* where *Y* represents super special product. We continued on until we arrived at the fixed point (a limit cycle) which formed a binary pair, named as the super hidden pattern of the dynamical super system. The resultant of every state vector gave way to the super hidden pattern always representing a fixed point. If we get the resultant for every state vector to be a fixed point in any dynamical system it means that the problem under study does not yield any changes in due course of time; namely, based on the opinion of the experts the problems are time independent; furthermore the views are not flexible.

Those who do not invest enough in R&D will have an increasingly smaller market and their profit will be also becoming smaller. In the medium or long term, such companies are bound in the first phase to decline then to extinction. Before the extinction the decline is manifested, which can be presented in three ways.

In the last chapter we presented the free mayor optimizing solutions for a company, highlighting the situations when the sales price is considered variable and the quantity sold constant in time and when the sales price is constant and quantity sold is variable.

The theoretical optimization part allows the determination of duration of an R&D cycle and the optimal number of cycles per a given range, while the numerical values in the graphs are for the qualitative emphasis of the curves forms.

From the previous relations ((1)–(20)), we find that the number of products sold to which innovation must be brought is greater than the number of products sold at *N*_pc_. The maximum is obtained if the innovation is introduced in the decline of sales at a distance of *N*_pc_ variable with investment in R&D, *C*_cd_ and with the *G* slope representing the decreasing sales. We would like to note that these models are absolutely original.

This paper contributes to the elaboration of solutions for the improvement of regional research and development through FRM model, useful to all entities (companies, organizations, and institutions) concerned with basic, applied research and experimental development.

## Figures and Tables

**Table 1:** The fuzzy super matrix.

		R1	R2	R3	R4	R5	R6	R7	R8	R9	R10	R11	R12
Q1	Q1.1	0	0	0	0	0	0	0	0	0	0	1	0
	Q1.2	0	0	0	1	1	0	0	0	1	1	0	0
	Q1.3	0	0	0	0	0	1	1	0	0	0	0	0
	Q1.4	0	0	1	0	0	0	0	0	0	0	0	0
	Q1.5	0	1	0	0	0	0	0	0	0	0	0	0
	Q1.6	1	0	0	0	0	0	0	1	0	0	0	1

Q2	Q2.1	1	0	1	0	1	1	0	1	0	0	0	0
	Q2.2	0	1	0	1	0	0	1	0	1	1	1	1

Q3	Q3.1	0	1	1	1	1	1	0	0	1	0	1	1
	Q3.2	1	0	1	1	0	0	0	0	0	1	1	1
	Q3.3	0	1	0	0	0	0	0	0	0	1	0	1
	Q3.4	0	1	1	1	0	0	0	0	0	0	0	0

Q5	Q5.1	0	0	0	0	0	1	1	0	0	0	0	0
	Q5.2	1	1	0	0	0	1	0	0	1	0	0	0
	Q5.3	0	0	0	1	1	1	0	0	0	1	0	1
	Q5.4	0	0	0	1	0	0	0	0	0	0	0	0
	Q5.5	0	0	0	0	0	0	0	0	0	0	0	0

Q6	Q6.1	0	0	0	0	0	0	0	0	0	0	1	0
	Q6.2	1	1	1	1	1	1	0	0	1	0	0	0
	Q6.3	0	0	0	0	0	0	1	0	0	1	0	1
	Q6.4	0	0	0	0	0	0	0	1	0	0	0	0

Q7	Q7	1	0	1	1	1	1	0	1	1	1	1	1

Q13	Q13	0	0	0	1	0	0	0	0	0	1	0	0

Q15	Q15.1	0	0	0	0	0	0	0	1	1	0	0	0
	Q15.2	0	0	0	1	0	0	0	1	0	1	1	1
	Q15.3	0	0	0	0	0	0	0	0	1	0	0	1
	Q15.4	1	0	0	0	1	1	0	0	0	0	0	0
	Q15.5	0	0	0	0	0	0	0	1	0	0	0	0
	Q15.6	0	0	1	0	0	0	0	1	0	0	0	0

Q17	Q17.1	1	0	0	0	1	1	0	0	0	1	0	0
	Q17.2	1	0	0	1	0	0	0	0	0	0	0	0
	Q17.3	1	0	1	1	1	1	0	1	0	1	1	1
	Q17.4	1	0	1	1	1	1	0	1	1	0	1	0

Q18	Q18.1	1	0	1	1	1	1	0	0	1	0	0	1
	Q18.2	1	0	0	0	1	1	0	0	0	1	0	0
	Q18.3	1	0	0	0	1	1	0	1	0	0	1	0

Q19	Q19	1	0	1	1	1	1	0	1	1	1	1	1
